# The ubiquinone synthesis pathway is a promising drug target for Chagas disease

**DOI:** 10.1371/journal.pone.0243855

**Published:** 2021-02-04

**Authors:** Takeshi Nara, Yukari Nakagawa, Keiko Tsuganezawa, Hitomi Yuki, Katsuhiko Sekimata, Hiroo Koyama, Naoko Ogawa, Teruki Honma, Mikako Shirouzu, Takehiro Fukami, Yuichi Matsuo, Daniel Ken Inaoka, Kiyoshi Kita, Akiko Tanaka

**Affiliations:** 1 Faculty of Pharmacy, Iryo Sosei University, Iwaki, Fukushima, Japan; 2 RIKEN Center for Biosystems Dynamics Research, Tsurumi, Yokohama, Japan; 3 RIKEN Center for Sustainable Resource Science, Wako, Saitama, Japan; 4 RIKEN Program for Drug Discovery and Medical Technology Platforms, Wako, Saitama, Japan; 5 School of Tropical Medicine and Global Health, Nagasaki University, Nagasaki, Japan; University of East Anglia, UNITED KINGDOM

## Abstract

Chagas disease is caused by infection with the protozoan parasite *Trypanosoma cruzi* (*T*. *cruzi*). It was originally a Latin American endemic health problem, but now is expanding worldwide as a result of increasing migration. The currently available drugs for Chagas disease, benznidazole and nifurtimox, provoke severe adverse effects, and thus the development of new drugs is urgently required. Ubiquinone (UQ) is essential for respiratory chain and redox balance in trypanosomatid protozoans, therefore we aimed to provide evidence that inhibitors of the UQ biosynthesis have trypanocidal activities. In this study, inhibitors of the human COQ7, a key enzyme of the UQ synthesis, were tested for their trypanocidal activities because they were expected to cross-react and inhibit trypanosomal COQ7 due to their genetic homology. We show the trypanocidal activity of a newly found human COQ7 inhibitor, an oxazinoquinoline derivative. The structurally similar compounds were selected from the commercially available compounds by 2D and 3D ligand-based similarity searches. Among 38 compounds selected, 12 compounds with the oxazinoquinoline structure inhibited significantly the growth of epimastigotes of *T*. *cruzi*. The most effective 3 compounds also showed the significant antitrypanosomal activity against the mammalian stage of *T*. *cruzi* at lower concentrations than benznidazole, a commonly used drug today. We found that epimastigotes treated with the inhibitor contained reduced levels of UQ_9_. Further, the growth of epimastigotes treated with the inhibitors was partially rescued by UQ_10_ supplementation to the culture medium. These results suggest that the antitrypanosomal mechanism of the oxazinoquinoline derivatives results from inhibition of the trypanosomal UQ synthesis leading to a shortage of the UQ pool. Our data indicate that the UQ synthesis pathway of *T*. *cruzi* is a promising drug target for Chagas disease.

## Introduction

Chagas disease (CD) is caused by infection with the protozoan parasite *Trypanosoma cruzi* (*T*. *cruzi*) transmitted through the feces of blood-sucking triatomine bugs, and is endemic in Central and South America regions that encompass the habitat of the triatomine bug. The parasite is also transmitted by human blood, blood products, or oral and congenital routes, therefore, it raises serious public health concerns in non-endemic countries that are accepting an increasing number of Latin American immigrants [1]. The estimated number of people worldwide infected with *T*. *cruzi* is 6 to 7 million [2]. The clinical course of CD varies, but often comprises an acute phase and a chronic phase. Approximately 30–40% of chronically infected patients develop cardiomyopathy and/or digestive alteration 10–30 years after infection [1].

Currently no vaccines for CD are available, and the present treatment of CD is based on the nitroheterocyclic compounds nifurtimox and benznidazole (BNZ). They are effective in the acute infection phase but show limited efficacy in the chronic phase. Furthermore, their severe adverse effects often interrupt the therapeutic protocol [3, 4]. Thus, development of effective new drugs is urgently required [5].

Ubiquinone (UQ), also known as coenzyme Q, is a lipophilic molecule composed of a benzoquinone and a long polyisoprenoid side chain. It is an essential electron mediator of respiratory chain and redox regulation in various organisms including trypanosomatids [6–8]. Lai *et al*. found that UQ depletion triggered the death of *Trypanosoma brucei* (*T*. *brucei*), a causative agent of African trypanosomiasis [9]. Treatment of the bloodstream form of *T*. *brucei* with 1-[(*n*-oct-1-ylamino)ethyl]1,1-bisphosphonic acid, an inhibitor of solanesyl-diphosphate synthase (SPPS) [10], led to depletion of the trypanosomal UQ pool, and UQ_10_ supplementation to the medium rescued the cell death [9]. These observations indicated that the inhibitor of SPPS suppressed *de novo* biosynthesis of the polyisoprenoid side chain of UQ and the resulting UQ pool depletion caused *T*. *brucei* killing.

In *T*. *cruzi*, the SPPS inhibitor bisphosphonates have been reported to kill *T*. *cruzi*, thus, the enzymes involved in the UQ biosynthesis pathway can be considered essential for survival. Twenty years ago, Urbina *et al*. firstly reported that a bisphosphonate derivative suppressed the proliferation of *T*. *cruzi* both *in vitro* and *in vivo* [11]. Later, Szajnman *et al*. synthesized various bisphosphonate derivatives with trypanocidal activity against *T*. *cruzi*, and also reported their inhibition against both farnesyl pyrophosphate synthase (FPPS) and SPPS, two enzymes involved in isoprenoid biosynthesis [10, 12–15]. Ferella *et al*. cloned *T*. *cruzi* SPPS, and showed that it catalyzes production of an isoprenyl chain with nine isoprene units, which corresponds well to the presence of UQ_9_ as its major UQ species, and supported the importance of FPPS and SPPS in *T*. *cruzi* [16].

The UQ biosynthesis pathway of *T*. *cruzi* is not fully documented, but that of mammalian has been extensively studied [17–19]. A central regulatory protein in the system is coenzyme Q biosynthesis protein 7 (COQ7) [20–22], a hydroxylase that converts demethoxy-UQ_10_ (DMQ_10_) to demethyl-UQ_10_ [23–25]. The *coq7* gene of *T*. *cruzi* was cloned and proven to have high homology to the human *coq7* gene [26]. Therefore, human COQ7 (hCOQ7) inhibitors [27, 28] are expected to cross-react with and inhibit trypanosomal COQ7 (tCOQ7).

To provide the evidence that inhibitors of the UQ biosynthesis enzymes have trypanocidal activities, we tested trypanocidal activity of clioquinol (CQ) against the mammalian stage of the parasite. CQ had been utilized as an oral intestinal amebicide and withdrawn from the market in the 1970s [29]. CQ has complex biological activities due to its metal chelating activity, and is recently paid attention for application to neurodegenerative disorders, like Alzheimer’s diseases, and cancers [30]. CQ also inhibits COQ7 in mammalian cells and nematodes, and its metal chelating activity is suggested to be involved in the activity [27]. Our preliminary experiments suggested the trypanocidal activity of CQ, as good as BNZ. Therefore, we have intended to study antitrypanosomal activity of other hCOQ7 inhibitors.

We have recently demonstrated that pyrazole derivatives inhibit hCOQ7 activity [28]. In the preliminary experiments, we selected the most active pyrazole hCOQ7 inhibitor, *1-(5-Chloropyridin-2-yl)-3-phenyl-1H-pyrazol-5-ol*, and examined its trypanocidal activity toward the mammalian stage of the parasite. The pyrazole hCOQ7 inhibitor showed only weak trypanocidal activity, which could not meet the criteria as a lead compound for development of chemotherapeutics.

In this study, we show that the oxazinoquinoline derivatives, newly selected potent hCOQ7 inhibitors, have antitrypanosomal activity. We also provide evidence that our oxazinoquinoline derivatives inhibit tCOQ7, leading to UQ depletion in the epimastigotes. We conclude that the UQ synthesis pathway is a promising drug target for CD therapeutics.

## Materials and methods

### Chemicals

In the cell-based screening of hCOQ7 inhibitors, compounds were obtained from the Open Innovation Center for Drug Discovery at the University of Tokyo, Japan (http://www.ocdd.u-tokyo.ac.jp/). Compound **1** (6-chloro-3-(1-phenylethyl)-3,4-dihydro-2*H*-[1,3]oxazino[5,6-*h*]quinoline) was purchased from LifeChemicals, Inc. (Niagara-on-the-Lake, Canada), compound **2** (6-chloro-3-(2-phenylethyl)-3,4-dihydro-2*H*-[1,3]oxazino[5,6-*h*]quinoline) from Enamine (Kyiv, Ukraine), and compound **3** (6-chloro-3-[2-(3,4-dimethoxyphenyl)ethyl]-3,4-dihydro-2*H*-[1,3]oxazino[5,6-*h*]quinoline) from Princeton BioMolecular Research, Inc. (Monmouth Junction, NJ). Compounds **1**, **2** and **3** showed a single peak by high-pressure liquid chromatography (HPLC) and were estimated as 99, 97 and 100% pure, respectively (S1 Fig). These compounds were dissolved in DMSO and stored at -30°C.

Structure and purity of the compounds were confirmed by ^1^H-NMR, mass spectrometry and UPLC. ^1^H-NMR spectra of compounds were recorded with tetramethylsilane as an internal standard using JEOL JNM-Ex 270 MHz spectrometer (Tokyo, Japan). Results of compound **1** were as follows: ^1^H-NMR (δ 8.96–8.98 (1H, m), 8.48–8.52 (1H, m), 7.49–7.54 (1H, m), 7.27–7.35 (5H, m), 7.13 (1H, s), 5.45–5.50 (1H, m), 5.17 (1H, d, J = 10.5 Hz), 4.27 (1H, d, J = 17.3 Hz), 4.03–4.10 (1H, m), 3.82 (1H, d, J = 17.3 Hz), 1.53 (3H, d, J = 6.8 Hz)), Mass: EI, m/e = 324 (M^+^).

### Human cell culture

Human cervix epidermoid carcinoma HeLa cells and human normal fibroblast WI-38 cells (a diploid human cell line composed of fibroblasts derived from lung tissue of a female fetus) were obtained from the RIKEN BioResource Research Center (Ibaraki, Japan). The cells were cultured in Dulbecco's Modified Eagle's Medium (GIBCO, Carlsbad, CA) containing 10% fetal bovine serum (FBS), penicillin (5,000 U/ml) and streptomycin (5 mg/ml), at 37°C in a 5% CO_2_ atmosphere. The cells were pre-cultured overnight and then subsequently co-cultured with each compound or 0.5% DMSO as a control.

The effects of compounds on the growth of human cell cultures were evaluated by the CellTiter-Glo 2.0 assay (Promega, Madison, WI) unless otherwise indicated. Various concentrations (0.1–30 μM) of the test compounds were added to the cells pre-cultured overnight in a 96-well culture plate. The cells were cultured for a further 4 days, and their viabilities were determined.

### Parasites

The Tulahuen strain of *T*. *cruzi* was gifted by Dr. Yoshimasa Kaneda (Tokai University School of Medicine) and used throughout the study [31, 32]. Epimastigotes were cultured in liver infusion tryptose (LIT) medium (No. 1029, ATCC medium formulations) supplemented with 10% FBS and 10 μg/ml of hemin (Sigma-Aldrich, St. Louis, MO) in tightly capped 25 cm^2^ culture flasks at 27°C. The mammalian stages of *T*. *cruzi* were maintained using mouse embryonic 3T3-SWISS albino fibroblasts (Health Science Research Resources Bank, Tokyo, Japan) as described [33].

To establish a luciferase-expressing *T*. *cruzi* for evaluation of the parasite growth we adapted the Gateway recombination system (Life Technologies, Carlsbad, CA) to a parasite expression vector, pTREX [34]. Briefly, the gene for modified firefly luciferase (*luc2*) was PCR-amplified using a pGL4.10 plasmid (Promega) and the primers (sense; 5'-CACCATGGAAGATGCCAAAAACATTAAGAAGGGC-3', antisense; 5'-GCCCTTCTTGGCCTTAATGAGAATCTCG-3') and the PCR product was cloned in pENTR/D-TOPO (Promega). The *luc2* gene was further cloned in a pTREX plasmid and introduced in epimastigotes as described [33]. The epimastigotes harboring the recombinant plasmid were selected with G418, cloned by limiting dilution, and used.

### Antitrypanosomal activity

In the experiments, epimastigotes were cultured at an initial cell density of 2 × 10^6^ cells/ml in glucose-free LIT medium supplemented with various concentrations (0.1–100 μM) of the compounds for 4 days. Live parasites were counted by microscopy. Alternatively, we used a luciferase-expressing *T*. *cruzi* for evaluation of the parasite growth. The luciferase activity was measured using the Picagene LT-2.0 detection reagent (Toyo Ink Group, Tokyo, Japan) and a luminometer (Infinite 200 PRO, Tecan Ltd., Männedorf, Switzerland). The cell numbers were determined from the chemiluminescence intensity, which showed a precise linear correlation with the cell number between 10^1^ and 10^7^. The epimastigote viability calculated from the luminescence intensity was fitted using biphasic non-linear regression or asymmetric non-linear regression (four parameters) using Prism 8 (GraphPad Software, La Jolla, CA) to calculate EC_50_ values.

Human WI-38 cells were inoculated in each well of a 24-well culture plate at a cell density of 1.5 × 10^4^/well. After 24 h incubation, 4.5 × 10^4^ trypomastigotes/well were added and cultured for a further 4 days in the presence of the test compounds (0.3–10 μM). The infected cells were washed with PBS to remove free parasites and stained with Diff-Quik solution (Sysmex, Kobe, Japan). Each image was captured using an optical microscope (Axioplan2, Carl Zeiss AG, Oberkochen, Germany) equipped with a digital camera (EOS Rebel T5i, Canon Inc., Tokyo, Japan) via a mount adaptor (NY-VS, Micronet Inc., Saitama, Japan). Host cells and amastigotes inside the host cells were counted manually as previously described [35]. The typical images were shown in S2 Fig.

### Analysis of quinones

Inhibitory activity of a compound on hCOQ7 enzyme activity was determined using HeLa cells, because UQ synthesis activity of human cells varies from strain to strain and HeLa cells have high activity and fit for the cell-based evaluation of hCOQ7 activity [28]. HeLa cells (2 × 10^5^ cells/well in a 6-well plate or 4 × 10^5^ cells/well in a 60 mm culture dish) were inoculated and cultured overnight, and then the test compounds (1–5 μM) were added and co-cultured for a further 2 days. Control samples were co-cultured with 0.5% DMSO. In most cases, four culture wells were prepared for each compound, of which two were used for quinone analysis and the others for protein determination. The total protein concentration was determined by the BCA Protein Assay Kit (TaKaRa, Shiga, Japan).

To analyze quinone contents harvested HeLa cells (10^4^–10^6^ cells) or epimastigote cells (1 × 10^7^ cells) were suspended in phosphate buffered saline (PBS) containing 5% K_3_Fe(CN)_6_. After addition of 120 ng UQ_9_ (for human samples) or UQ_10_ (for trypanosomal and mouse samples) as an internal standard to quantify quinone amounts by HPLC analysis, the quinone fractions were extracted from the cell suspension by vigorous mixing in seven volumes of ethanol/*n*-hexane (2:5, v/v) solution for 10 min, and then centrifuged at 9,100 × g for 5 min at room temperature. The supernatants were pooled and dried in an evaporation device, and the residues were dissolved in 30 μl methanol and analyzed by HPLC with a UV detector (275 nm) (UV-2075, JASCO, Tokyo, Japan). Each 10 μl aliquot of the quinone fraction was injected to a reverse phase HPLC column (CAPCELL PAK C18IF S2, 2 μm, 2.0 × 50 mm, OSAKA SODA, Osaka, Japan), and eluted under isocratic conditions (0.15 ml/min, 30°C), with diisopropyl ether/methanol (10:90, v/v for human extracts; 2:98, v/v for trypanosomal and mouse extracts) as described previously [36, 37].

### *In silico* screening of compounds with similar structure

Compounds were selected by 2D and 3D ligand-based similarity searches. We calculated the Tanimoto coefficient between compound **1** and each of the 6,868,468 commercially available compounds on the basis of 2D fingerprints (MACCS public keys, ECFP4, FCFP4 and GpiDAPH3) and 3D shape metrics (ComboScore) with the software Pipeline Pilot 2017 (Dassault Systèmes, San Diego, CA), MOE 2015.1001 (Chemical Computing Group, Montreal, Quebec, Canada), and ROCS 3.2.0.4 (OpenEye Scientific Software, Santa Fe, NM.) [38]. Finally, 38 compounds showing high Tanimoto coefficient values from each metric were selected and purchased.

### Liquid chromatograph/time-of-flight mass spectrometry

Liquid chromatograph/time-of-flight mass spectrometry (LC-TOF/MS) analysis of quinone was performed by Kaneka Corporation (Osaka, Japan). In short, a time-of-flight mass spectrometer (TripleTOF 6600 system, SCIEX, Tokyo, Japan) equipped with a DuoSpray ion source was coupled to a liquid chromatographic system (Nexera X2, Shimadzu, Kyoto, Japan). Detection of tandem mass analysis was implemented over a mass range of 100–1,000 (*m/z*). Ionization was performed using an atmospheric pressure chemical ionization (APCI) probe (positive polarity mode). The manufacturer’s supplied software (Analyst TF software) was used for post-acquisition analysis.

### Statistical analyses

Statistical analysis and regression were conducted with GraphPad Prism 8/9 software (GraphPad Software, Inc., San Diego, CA). All data are presented as mean ± standard deviation, and numbers of independent replication were mentioned in the legends.

## Results and discussion

### An oxazinoquinoline hCOQ7 inhibitor has antitrypanosomal activity

In our previous study, hCOQ7 inhibitors have been selected as the compounds that inhibit hCOQ7 hydroxyl activity and thereby lead to accumulation of its substrate molecule, DMQ_10_ in HeLa cells [28]. In the study, 36 test compounds were excluded from analysis of quinones because of their inhibitory effects on the growth and adhesion of HeLa cells. In this study, we analyzed their inhibitory activities on quinone synthesis. Among them, only one compound, (6-chloro-3-[2-(1-cyclohexen-1-yl)ethyl]-3,4-dihydro-2*H*-[1,3]oxazino[5,6-*h*]quinoline) caused substantial accumulation of DMQ_10_. The levels of DMQ_10_ in the cells treated with the compound at 3 μM for 2 days increased and reached more than 20% of the total quinone content, indicating its strong inhibition against hCOQ7. However, we found that both of the 2 lots of the compound purchased from different companies for further study had two major HPLC peaks, suggesting the possibility of a mixture of two different chemicals. Thus, a structurally similar compound, (6-chloro-3-(1-phenylethyl)-3,4-dihydro-2*H*-[1,3]oxazino[5,6-*h*]quinoline) (compound **1,** see Fig 3), was purchased from the companies. We confirmed the compound **1** showing a single peak by HPLC with more than 98% purity and therefore, used it for further analyses (S1 Fig).

**Fig 1:**
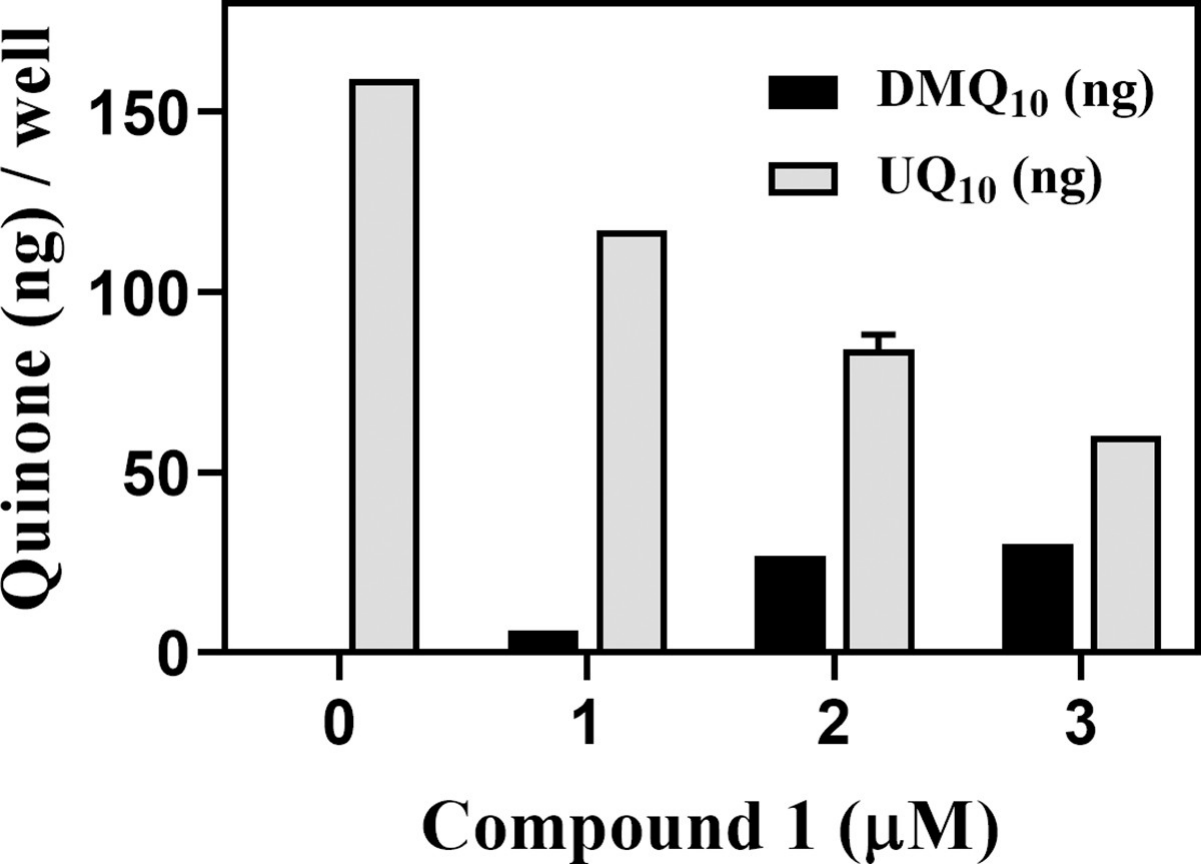
Quinone amounts in HeLa cell cultures treated with compound 1. HeLa cells (4 × 10^5^ cells/well) were cultured in the presence of compound **1** for 2 days at the indicated concentrations, and the amounts of UQ_10_ and DMQ_10_ in the harvested cells were analyzed. The graph is representative of two independent experiments performed in duplicate quantification. The quinone amounts (ng) / well are presented as mean ± standard deviation.

The oxazinoquinoline derivative, compound **1**, was tested for its inhibitory effect on the hCOQ7 activity by detection of accumulation of DMQ_10_ in HeLa cells. In addition to the endogenous UQ synthesis activity, human cells can take up exogenous UQ [39], and the UQ synthesis activity varies widely depending on the cell line [28]. HeLa cells have the higher UQ synthesis activity and are appropriate to use for the cell-based assay for hCOQ7 activity [28]. Quinone fractions were prepared from HeLa cells treated for 2 days with compound **1**, and their quinone amounts were analyzed by HPLC (Fig 1). In the presence of 1 to 3 μM of the compound **1**, the culture extracts contained reduced amounts of UQ_10_ and increased amounts of DMQ_10_ in a dose-dependent manner. These observations indicated that compound **1** inhibited hCOQ7 in HeLa cells resulting in the reduction of UQ_10_ and accumulation of DMQ_10_. It is worth to note that compound **1** inhibited hCOQ7 activity at a much lower concentration compared with the effective concentrations (10–20 μM) of the previously reported pyrazole derivatives [28]. Therefore, we examined compound **1** for its trypanocidal activity.

**Fig 2:**
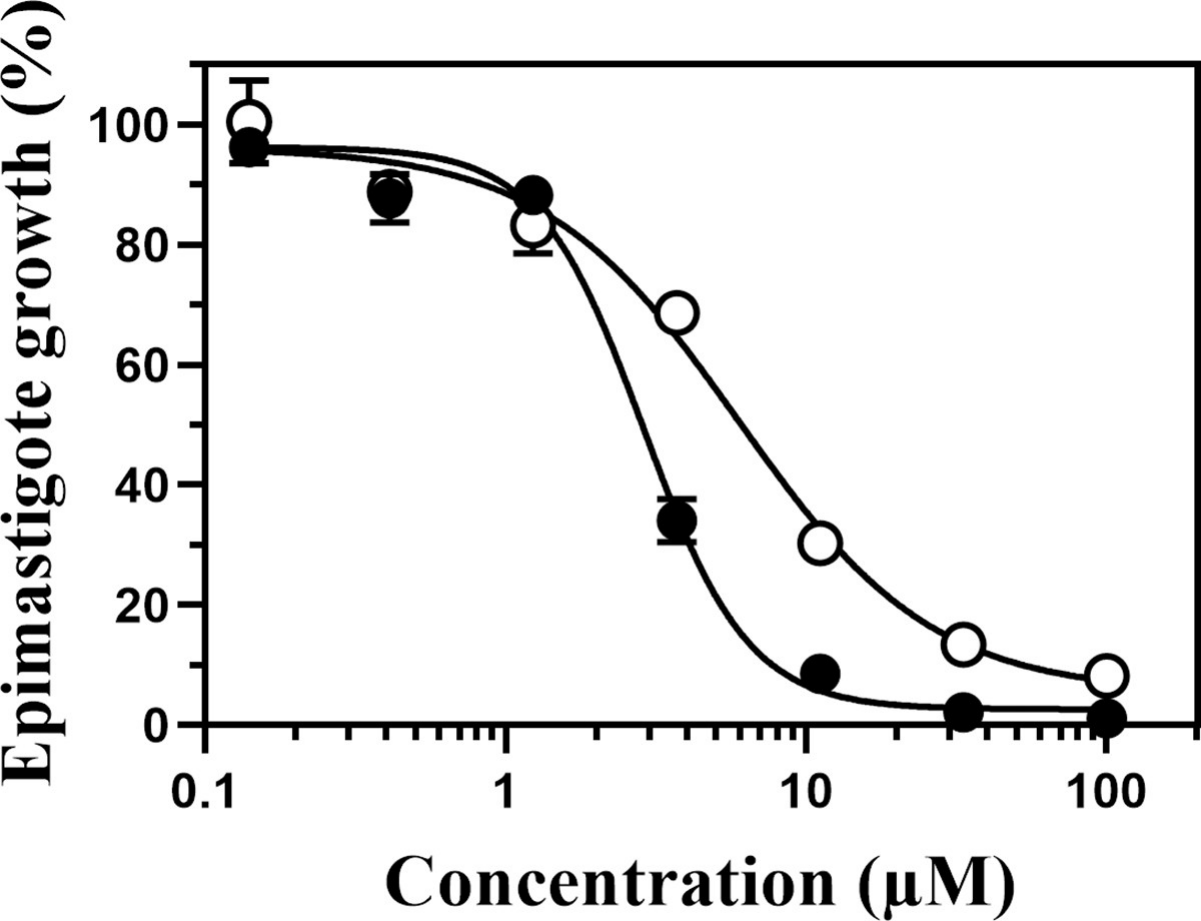
Growth of *T*. *cruzi* epimastigotes treated with compound 1 and BNZ. The luciferase-expressing epimastigotes (5 × 10^5^ cells/ml) were cultured for 4 days in a medium containing various concentrations of the compounds, and their numbers were determined by measuring the luciferase activity. The open circles indicate the results of BNZ treatment, and the closed circles indicate those of compound **1**. The typical results of three independent experiments are shown. Epimastigote growth was determined by triplicate quantification and is indicated as mean ± standard deviation.

The antitrypanosomal activity of compound **1** against *T*. *cruzi* epimastigotes was examined. The luciferase-expressing epimastigotes (5 × 10^5^ cells) were cultured in each well of a 96-well culture plate containing compound **1** or BNZ (as a control) at various concentrations. After treatment for 4 days, epimastigote numbers were determined by measuring the luminescence intensity of each well (Fig 2). Compound **1** killed the epimastigotes at lower concentrations than BNZ. The estimated IC_50_ values of compound **1** and BNZ were 2.4 ± 0.90 μM and 5.9 ± 0.06 μM, respectively (determined with non-linear 4 parameter regression, n = 3). Notably, compound **1** succeeded in killing off all epimastigotes at higher concentrations (> 33 μM), suggesting that hCOQ7 inhibitors are able to cause lethal effect on the epimastigotes.

### Oxazinoquinoline derivatives with similar structure have antitrypanosomal activity

Next, we investigated whether other oxazinoquinoline derivatives exhibit antitrypanosomal activity. Structurally similar compounds were selected by 2D and 3D ligand-based similarity searches by calculating the Tanimoto coefficients between compound **1** and each of the 6,868,468 commercially available compounds. Based on the calculation, 38 compounds with higher homology were purchased, and their trypanocidal activities against epimastigotes were tested *in vitro*. The parasite cells were inoculated into each well of a 96-well culture plate (5 × 10^5^ cells/ml) containing each test compound at 10 μM, and further cultured for 4 days. Treatment with compound **1**, a positive control, decreased epimastigote growth to 5.2 ± 2.00% after the treatment. Because epimastigotes without treatment propagated approximately four times under these experimental conditions, we regarded a compound that suppressed epimastigote viability to less than 25% as an active trypanocidal compound. Among the 38 compounds, 12 compounds were shown to be active, 17 compounds showed moderate antitrypanosomal effects, and 9 compounds were less effective. Compound **1** and 12 active compounds were identified as antitrypanosomal oxazinoquinoline derivatives (Table 1 and S1 Fig). All active compounds have the 6-chloro oxazinoquinoline structure as compound **1**. No compounds with the oxazinoquinoline structure were contained in the less active group, so that we concluded that the oxazinoquinoline structure is essential for antitrypanosomal activities (S1 Fig).

**Table 1:** List of the oxazinoquinoline derivatives effective on epimastigote growth.

Compound No.^a^	Epimastigote Viability (%)^b^
	** **
**1**	**5.2 ± 2.00**
**2**	**3.1 ± 0.09**
**3**	**3.5 ± 0.12**
**4**	**3.6 ± 0.11**
**5**	**6.3 ± 0.93**
**6**	**6.6 ± 0.04**
**7**	**6.9 ± 0.04**
**8**	**7.1 ± 0.27**
**9**	**7.1 ± 0.06**
**10**	**7.2 ± 0.17**
**11**	**10.6 ± 0.24**
**12**	**13.2 ± 0.07**
**13**	**14.1 ± 1.12**

^a^Purity of compounds determined by HPLC were as the following: 99% (**1**, racemate), 97% (**2**), 100% (**3**), 98% (**4**), 99% (**5**), 100% (**6**), 99% (**7**), 97% (**8**), 100% (**9**), 97% (**10**), 98% (**11**), 87% (**12**), 99% (**13**).

^b^All data are presented as mean ± standard deviation of duplicated results.

**Fig 3:**
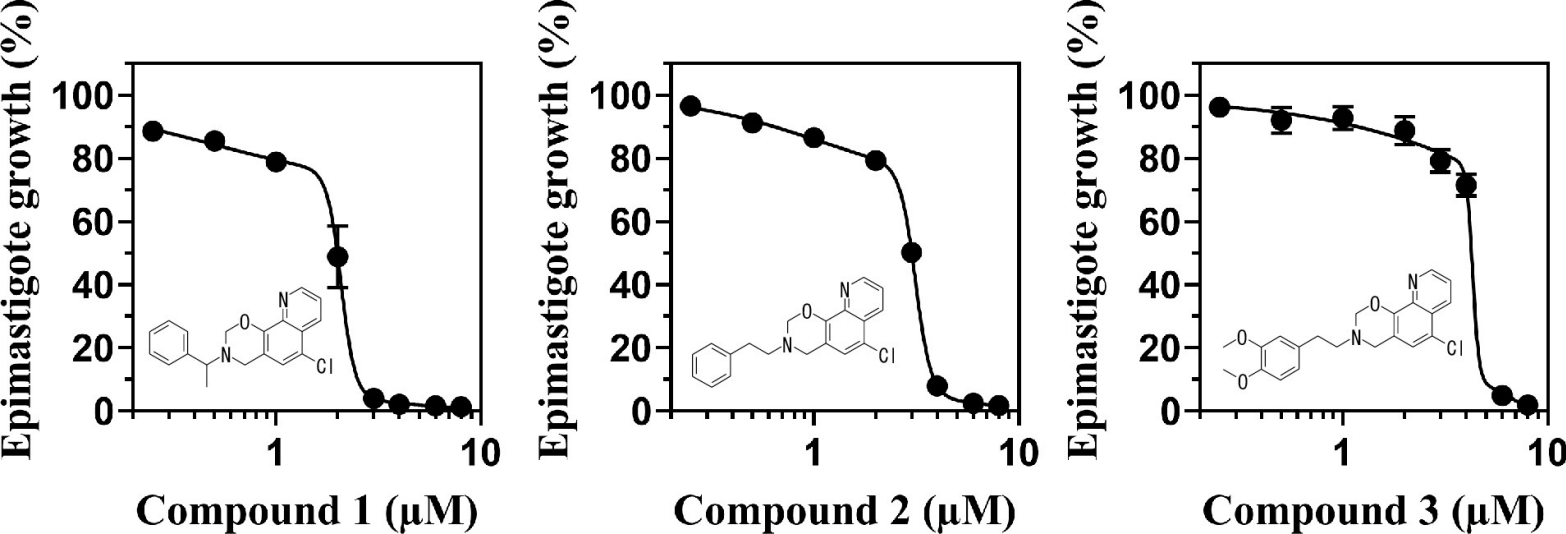
Inhibition of epimastigote growth by compounds 1–3. Dose-response effects of the compounds on epimastigote growth after a 4-day treatment are shown. The regression curves and EC_50_ values were calculated by biphasic non-linear regression using the software PRISM 8.4.1. The graph is representative of three independent experiments and the growth rates (%) are indicated as mean ± standard deviation.

Many of active oxazinoquinoline derivatives have a benzyl or a phenethyl substituent, so compounds **1**, **2**, and **3** with the higher activity were selected as representative compounds for further study (Fig 3). The EC_50_ values for epimastigote growth of compounds **1**, **2**, and **3** were 2.0 ± 0.04, 3.0 ± 0.01, and 4.2 ± 0.06 μM, respectively (determined with biphasic non-linear regression, n = 3), whereas the reference drug BNZ had an EC_50_ value of 6.0 ± 0.33 μM (determined with non-linear 4 parameter regression, n = 3). The plots for compounds **1–3** fitted well to biphasic non-linear regression curves, suggesting that the compounds suppressed epimastigote growth mildly at lower concentrations and then severely inhibited it at higher concentrations (Fig 3). Because *de novo* UQ synthesis is essential for *T*. *cruzi*, the gradual decreases of the cell number at the lower concentrations of the compounds might reflect growth retardation likely due to homeostatic perturbation caused by the inhibitors. At the higher concentrations, the UQ pool might be depleted to a lethal level, and it caused the death of the epimastigotes due to homeostasis disruption.

**Fig 4:**
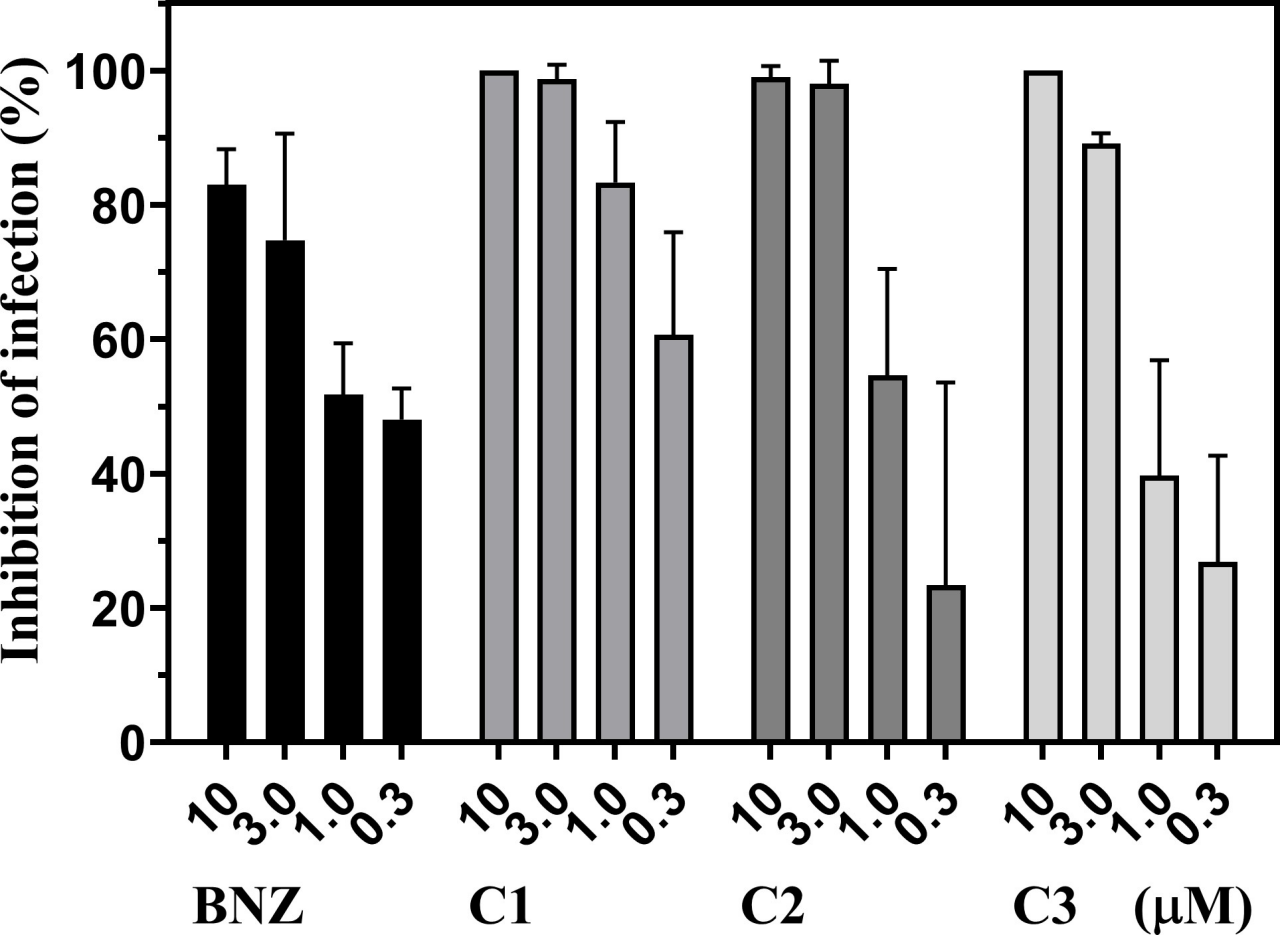
Inhibition of *T*. *cruzi* infection by compounds 1–3. Compounds were added to infected host cultures at the indicated concentrations. After 4 days, the infection rates were manually counted using three photos showing 235–607 total host cells and inhibition rates were calculated. The graph is representative of two independent experiments and indicates mean inhibition ± standard deviation.

We further examined to determine if these oxazinoquinoline derivatives had trypanocidal activity in the mammalian stage of the parasite. WI-38 cells were infected by trypomastigotes and cultured for 4 days in the presence of 10, 3, 1, and 0.3 μM of the test compounds. The infection rate was evaluated by microscopic observation of hundreds of cells after staining. Host cells and amastigotes inside the host cells were counted manually using three photo images for each sample (S2 Fig). Compounds **1–3** strongly inhibited the parasite infection at 10 and 3 μM, all of which was more effective than BNZ (Fig 4). Especially, no amastigotes were observed after 10 μM treatment of compounds **1** and **3**. It is worth to note that the total clearance of the *T*. *cruzi* infection is critical as a promising drug candidates for CD, as Cal *et al*. reported in the evaluation study of CYP51 inhibitors [40].

To know if the compounds inhibit not only the growth of the intracellular amastigotes but also the infectivity of trypomastigotes, we added the compounds one day after the infection. We did not find any difference of the status of infection between the simultaneous and subsequent additions of the compounds at the infection (S3 Fig). It indicated that the compounds affected viability of amastigotes, the mammalian stage of *T*. *cruzi*.

Since the metabolic half-life of UQ_9_ in rat is reported to vary between 2 and 5 days depending on the tissue evaluated [41], it is likely that inactivation of UQ synthetic enzyme(s) of *T*. *cruzi* by the inhibitors also results in the gradual decrease of the endogenous UQ pool during several days. Most of the infective trypomastigotes invade inside the host cells within 24 hours [42], therefore, our compounds may not affect the infectivity of trypomastigotes. The inhibitory effect of the compounds may be caused by their subsequent incubation in the infected culture and specific to the amastigote stage in our assay system.

As for cytotoxicity, WI-38 cells were treated with compounds **1**–**3** for 4 days, and their IC_50_ values were calculated (Table 2). Selectivity index indicated that compounds **1**–**3** specifically inhibited the infection of WI-38 cells with *T*. *cruzi*. As shown in Table 2, these compounds had mild cytotoxicity against human culture cells, it might be because that these compounds had been selected owing to their high similarity to compound **1**, a cytotoxic compound.

**Table 2:** Effects of compounds on amastigote infection and human culture cells.

Compound	Amastigote	WI-38	Selectivity index^c^
	Infection	Viability	
	IC_50_ (mM)^a^	IC_50_ (mM)^b^	
**1**	0.23 ± 0.03	6.0 ± 0.24	26.1
**2**	0.46 ± 0.16	8.0 ± 1.50	17.4
**3**	0.76 ± 0.31	8.1 ± 0.52	10.7
BNZ	0.43 ± 0.08	>30.0	-

^a^IC_50_ values were determined using the results of two independent experiments and calculated by non-linear regression using the software PRISM 9.0.0.

^b^IC_50_ values (n = 3) were calculated by non-linear regression using the software PRISM 9.0.0.

^c^Selectivity index = WI-38 IC_50_ / Amastigote infection IC_50_.

**Fig 5:**
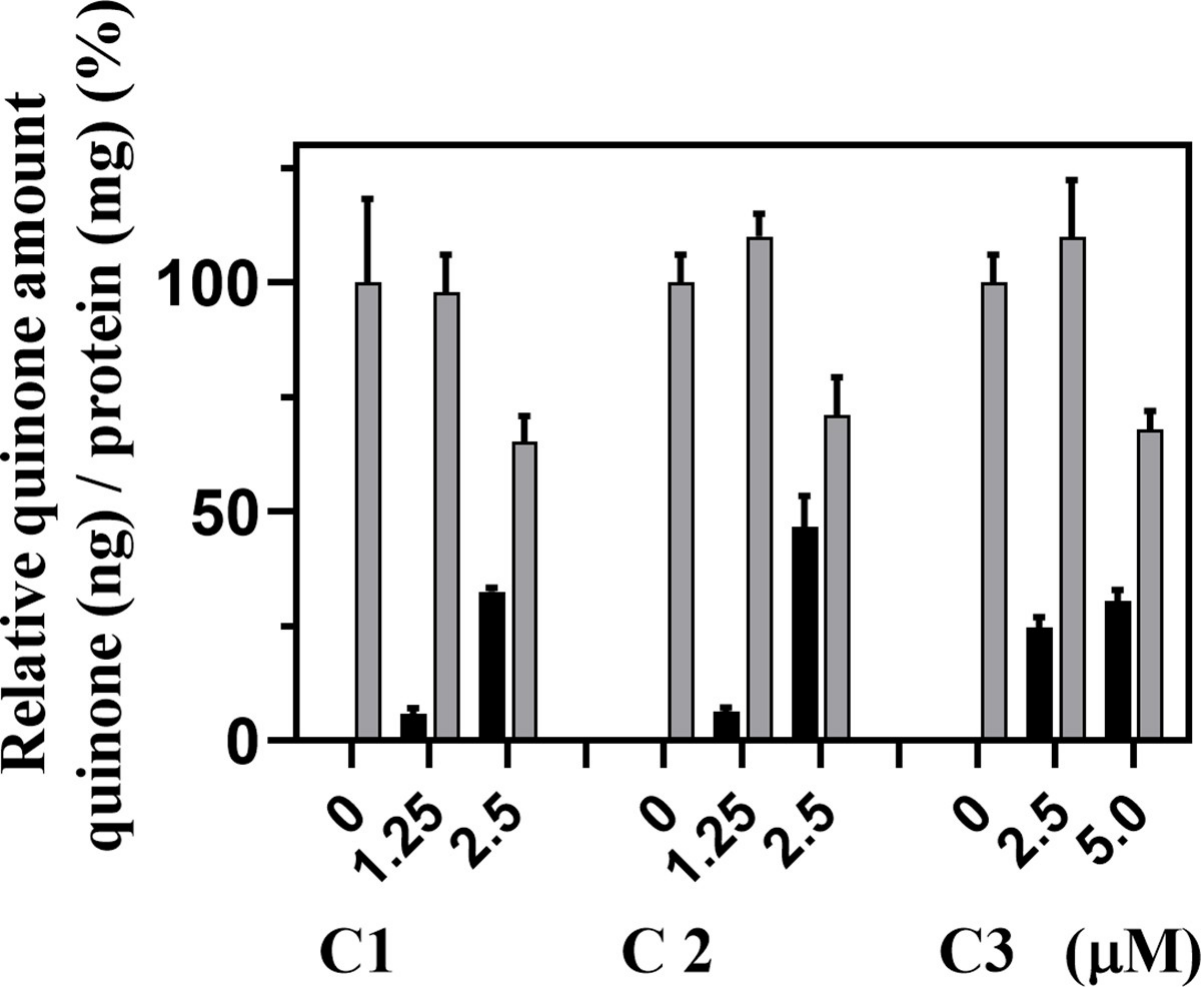
Quinone amounts in HeLa cells treated with compounds 1, 2, and 3. HeLa cells (2 × 10^5^ cells/well) were inoculated and pre-cultured for 1 day, and cultured for further 2 days in the presence of the compounds at the indicated concentrations. The amounts of UQ_10_ and DMQ_10_ (ng) / protein (mg) were determined and normalized with the UQ_10_ amount of each control culture. The gray bar indicates the value of UQ_10_, and the black bar indicates that of DMQ_10_. The typical results of three independent experiments are shown with mean ± standard deviation of duplicate results.

We consider that hCOQ7 inhibition itself does not cause cell death of human culture cells, because the mammalian cells have two UQ supplementation pathways: endogenous UQ synthesis [17], and exogenous UQ uptake [39]. Although, development of compounds specifically inhibit tCOQ7 is an attractive point to consider the oxazinoquinoline derivatives as drug candidates. Thus, we examined the hCOQ7 inhibition activity of the selected compounds by measuring the quinone amount (ng) per total protein amount (mg) in HeLa cells treated for 2 days (Fig 5). The cells treated with 2.5–5 μM of the compounds showed not only reduction of UQ_10_, but also accumulation of DMQ_10_. Because DMQ_10_ is the substrate of hCOQ7, these data indicate that the compounds inhibited hCOQ7 and resulted in suppressing UQ synthesis in the HeLa cells at 2.5 μM (compounds **1** and **2**) or 5 μM (compound **3**). Our analysis suggests that introduction of bulky substituents on the phenethyl group of oxazinoquinoline derivatives would weaken the inhibitory activity against hCOQ7, as observed with compound **3**.

When HeLa cells were treated with compound **1** at 1.25 μM, we detected the accumulation of DMQ_10_, whereas the amount of UQ_10_ was not clearly altered. We also found the increased levels of UQ_10_ by treatment with compounds **2** and **3** at lower concentrations (Fig 5). We examined the levels of hCOQ7 in HeLa cells treated with low concentrations of the compounds by Western blotting, and observed rather increased levels of hCOQ7 after the treatment for 3 days (S4 Fig). Although regulation of UQ synthesis via hCOQ7 is still an issue of debate, the complicated regulatory system is known to present in eukaryotes [23–25]. It is likely that the up-regulation of UQ synthesis, associated with enhanced expression of hCOQ7 protein, might occur by weak inhibition of hCOQ7 (S4 Fig). We have also observed the weak up-regulation of UQ synthesis in *T*. *cruzi* treated with compound **1** (Fig 8, discussed later).

### A reduced UQ pool is responsible for the antitrypanosomal activity of the oxazinoquinoline derivative

**Fig 6:**
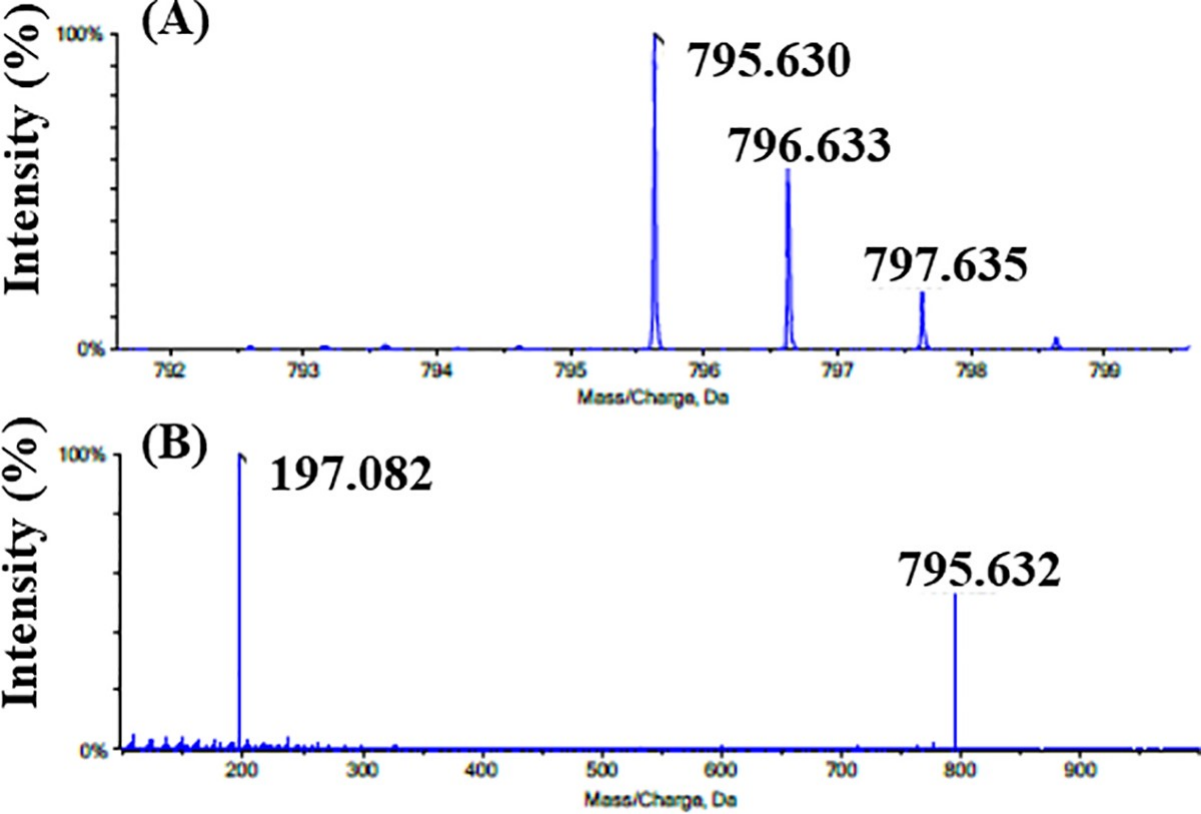
(A) The mass spectrum and (B) tandem mass spectrum of UQ_9_ contained in the epimastigote quinone fraction.

Taking their hCOQ7 inhibition activity and their killing activity against *T*. *cruzi* into consideration, these compounds are expected to cross-react with tCOQ7, suppress *T*. *cruzi* UQ synthesis, and thereby deplete the parasite UQ pool leading to cell death. To study the trypanosomal UQ biosynthesis, we analyzed the chemical structures of the quinone components in wild-type epimastigotes using an LC-TOF/MS method. The quinone fraction was extracted from 1 × 10^9^ epimastigotes and analyzed. The major UQ content from the control epimastigotes displayed an accurate molecular weight of m/z 795.630 with a molecular formula of C_54_H_82_O_4_, exactly corresponding to the molecular weight of UQ_9_ (Fig 6A). When the [M+H]^+^ was selected as the precursor ion for MS/MS, it further generated a fragment ion with m/z 197.082, which had a molecular formula of C_10_H_13_O_4_ derived from its quinone ring (Fig 6B). These LC-TOF/MS analyses indicated that the epimastigotes contained UQ_9_ as a major quinone component as reported previously [16]. Further analyses showed that very small amounts of UQ_8_, UQ_10_, and DMQ_9_ were also contained in the epimastigotes. The polyisoprenoid chain of the UQ molecule consists of nine isoprene units (UQ_9_) in *T*. *cruzi* and mice, and 10 units (UQ_10_) in human and bovine [17]. Despite the determined length of nine isoprene units, we also found low levels of UQ_10_ in *T*. *cruzi* epimastigotes. The existence of small amount of UQ_10_ was also reported in cells of mouse tissue [43]. Because the epimastigotes were cultured in a medium containing 10% FBS, the heterogeneity of the UQ species may suggest that epimastigotes are able to take up low levels of exogenous UQ_10_ from their culture medium.

**Fig 7:**
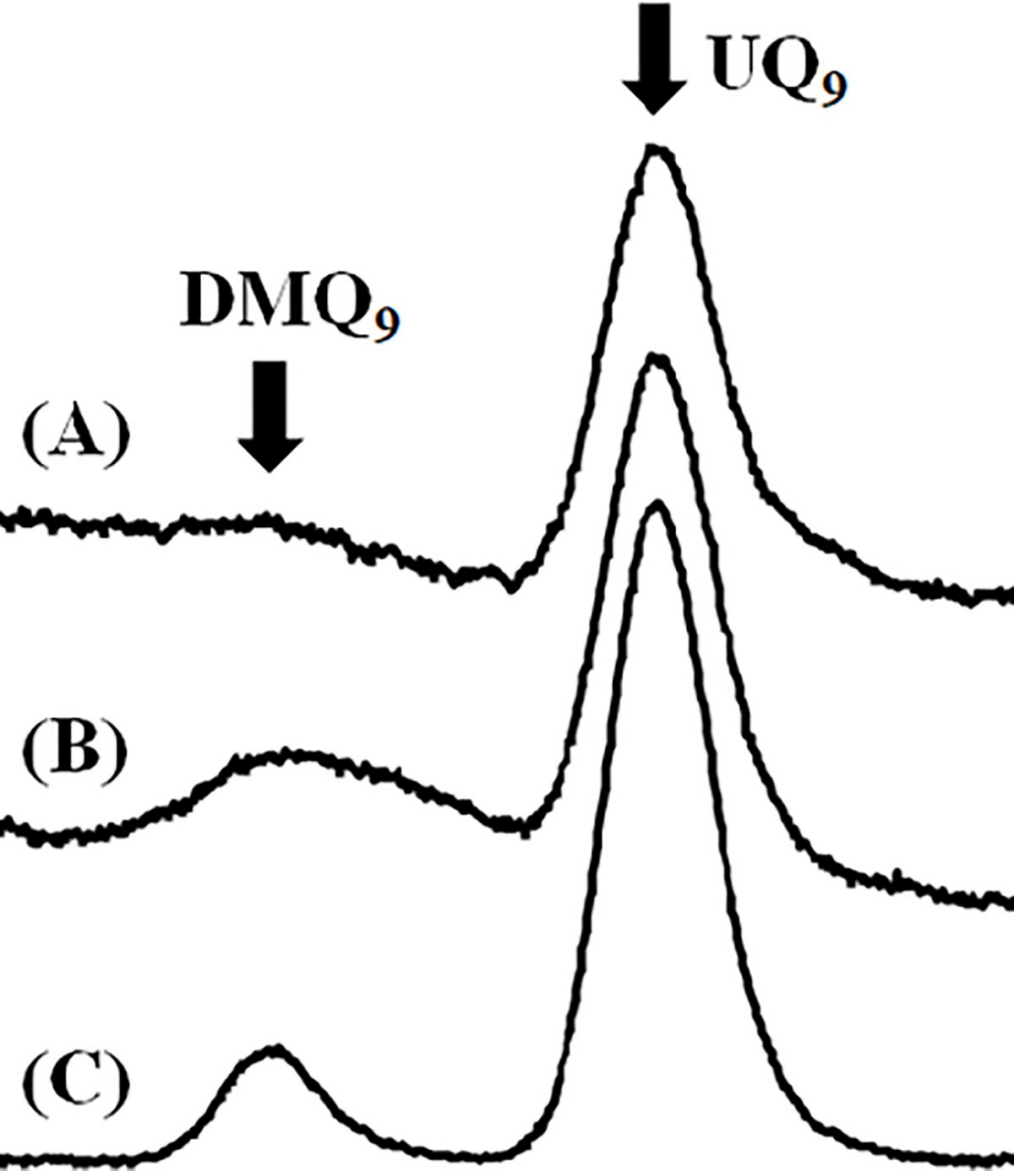
Quinone components of epimastigotes treated with or without compound 1. Representative chromatograms of quinone fractions from (A) control epimastigotes, (B) epimastigotes treated with 1 μM compound **1** for 4 days, and (C) RAW264.7 cells treated with 20 μM CQ for 1 day.

We examined if compound **1** affects trypanosomal UQ biosynthesis as in human cells. Quinone fractions extracted from wild-type epimastigotes treated with or without compound **1** were analyzed by HPLC, and examined. Fig 7A shows a representative HPLC spectrum of a quinone fraction extracted from wild-type epimastigotes, and Fig 7B shows that from epimastigotes treated with 1 μM compound **1** for 4 days. We compared these spectra with that obtained from the cells of a mouse macrophage-like cell line, RAW264.7 treated with CQ (Fig 7C). The quinone fractions of RAW264.7 cells treated with CQ were reported to contain both UQ_9_ and DMQ_9_ because CQ is a known inhibitor of mouse COQ7 [27]. Thus we prepared and analyzed the sample as a control, and identified the major peak for UQ_9_ and the minor peak corresponding to DMQ_9_ (Fig 7C). Comparing the three spectra, the control epimastigote fraction (Fig 7A) contained a major HPLC peak corresponding to UQ_9_ and a faint signal corresponding to DMQ_9_. The quinone fraction of the epimastigotes treated with 1 μM compound **1** (Fig 7B) showed two peaks: the major UQ_9_ peak and an increased broad peak corresponding to DMQ_9_. Our data clearly indicate that compound **1** cross-reacted with and inhibited tCOQ7 resulting in accumulation of its substrate molecule, DMQ_9_.

**Fig 8:**
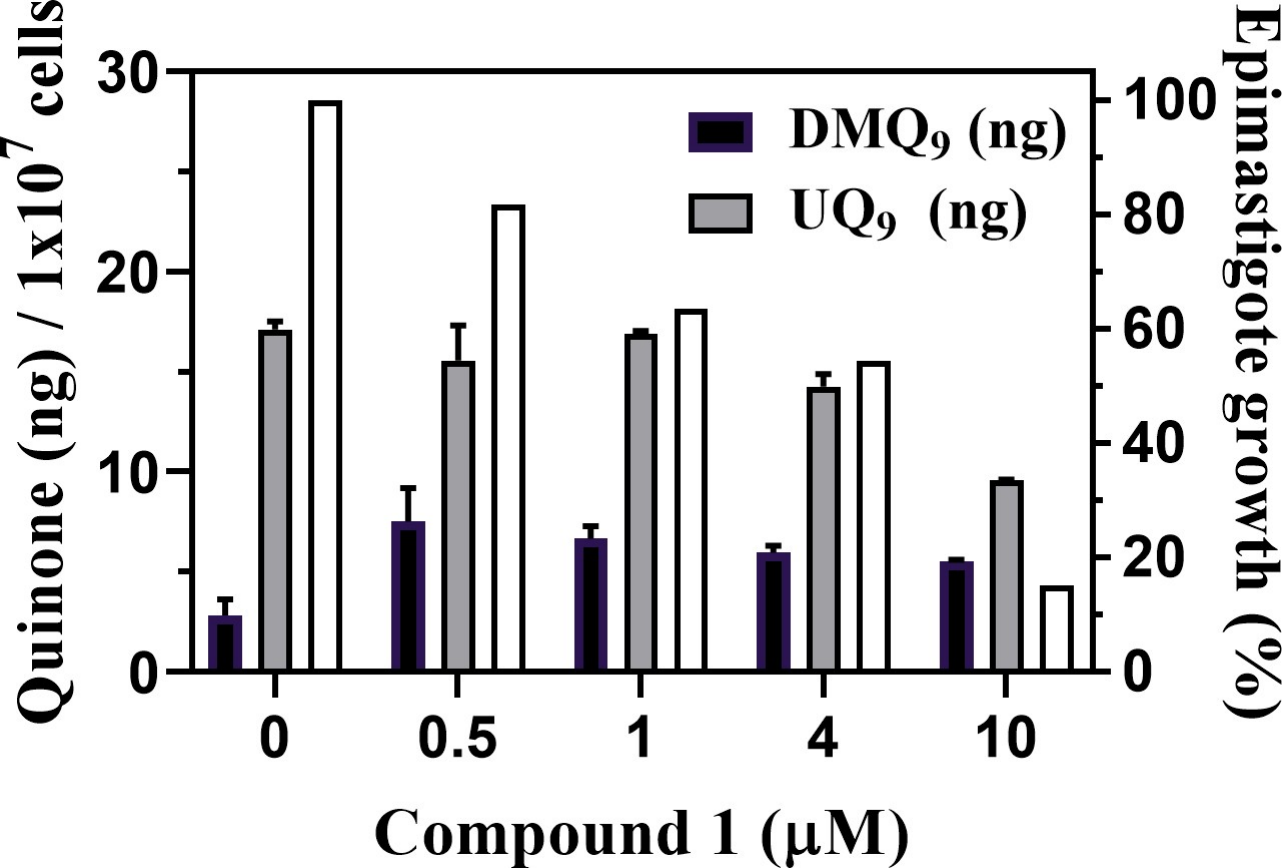
Quinone amounts in epimastigotes treated with compound 1. Quinone fractions were extracted from epimastigotes (1 × 10^7^ cells) treated with various concentrations of compound **1** for 4 days. The epimastigote growth (%) in each culture was calculated (white bars). The amounts of UQ_9_ (gray bars) and DMQ_9_ (black bars) were calculated by comparing their signal areas to that of the added internal standard, UQ_10_. The mean quinone amount (ng) / 10^7^ cells are indicated as mean ± standard deviation of duplicate measurements.

Then, the quinone components of the wild-type epimastigotes treated with various concentrations of compound **1** were quantified. After a 4-day co-culture, the cell densities of epimastigotes in each culture were counted (Fig 8, white bars), and their quinone fractions from 1 × 10^7^ cells were prepared and analyzed by HPLC. The amounts of quinones in the extracts (Fig 8, black and gray bars) were determined by comparing peak areas with those of the added internal standard, UQ_10_.

Notably, the amounts of DMQ_9_ in the epimastigotes were increased by the treatment with compound **1**, showing that the compound inhibited tCOQ7 and suppressed hydroxylation of DMQ_9_. In this case, DMQ_9_ accumulation did not further increase in cells treated with more than 1 μM of compound **1**, suggesting the possibility of additional activity of compound **1** inhibiting *de novo* DMQ_9_ synthesis. By comparison, when epimastigotes were treated with compound **1** at more than 1 μM, the amounts of UQ_9_ (gray bars in Fig 8) as well as the cell density (white bars in Fig 8) decreased in a dose-dependent manner. These results suggest that compound **1** inhibited trypanosomal UQ biosynthesis, leading to reduction of the UQ pool and subsequent cell death.

The UQ pool appears to be critical for redox homeostasis, since oxidation-reduction reaction in energy metabolism mediated by UQ yields intracellular reactive oxygen species [44], and the reduced form of UQ regenerates antioxidants in the cells [45, 46]. Further, the redox homeostasis systems are important drug targets for *T*. *cruzi* [47]. Inhibitors of trypanothione metabolism and free radical-producing drugs, such as naphthoquinones, nifurtimox and BNZ, have been extensively developed [3, 48–50]. Therefore, the reduction of the UQ pool along with cell division without *de novo* UQ supply is supposed to causes redox imbalance and subsequent cell death of *T*. *cruzi*.

In this experiment, the treatment with compound **1** at concentrations lower than 1 μM inhibited tCOQ7 and resulted in DMQ_9_ accumulation, but did not significantly affect the levels of UQ_9_ (Fig 8). It is likely that the increased levels of UQ by weak inhibition of tCOQ7 might occur due to increased expression of tCOQ7 protein, as observed with hCOQ7 in HeLa cells (Fig 5 and S4 Fig). This homeostatic perturbation by compound **1** might cause the gradual suppression of the parasite growth at the lower compound concentrations because *T*. *cruzi* epimastigotes have a finely orchestrated metabolic switch related to energy metabolism as Barison *et al*. reported [51].

**Fig 9:**
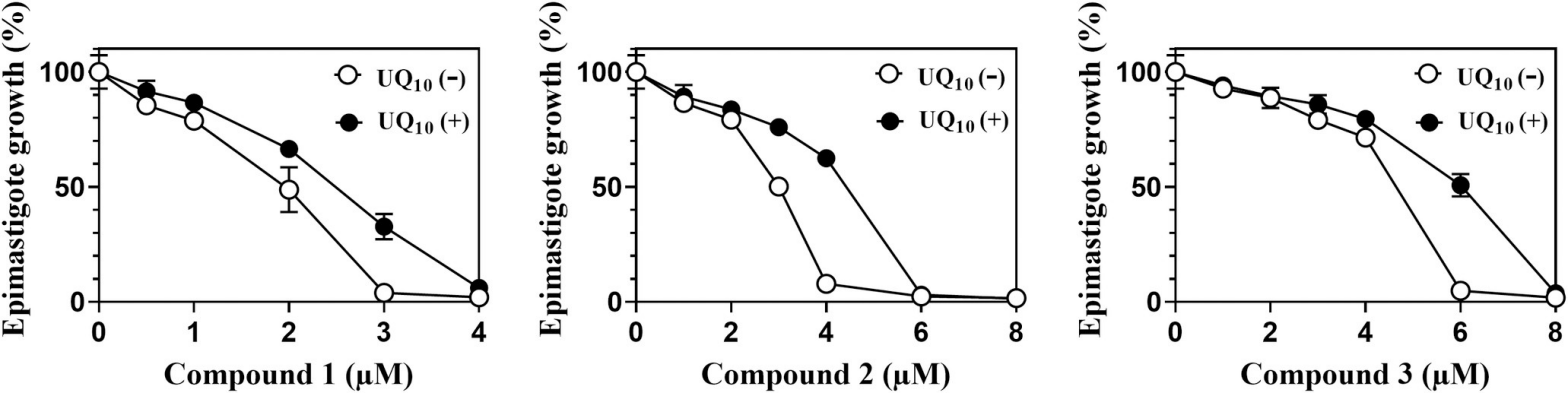
Addition of UQ_10_ weakened the trypanocidal activity of the oxazinoquinoline derivatives. Dose-response effects of the compounds on luciferase-expressing epimastigote growth were measured after a 4-day culture using the medium with (closed circles) or without (open circles) 30 μM UQ_10_. The typical results of two independent experiments are shown. Growth (%) is indicated as mean ± standard deviation of triplicate results.

We considered that the antitrypanosomal activity of our compounds was attributed to a shortage of the protozoan UQ pool, and therefore could be rescued by supplementation with UQ_10_ as reported previously for *T*. *brucei* [9]. The luciferase-expressing epimastigotes were treated with various concentrations of compounds **1–3** in the presence or absence of 30 μM UQ_10_ in the culture medium as an external UQ source (Fig 9). The impaired growth of the parasites was rescued by the treatment with 30 μM UQ_10_ at the higher lethal concentrations of the compounds, whereas little effect was observed at the lower non-lethal, but suppressive concentrations. We concluded that the oxazinoquinolines inhibited epimastigote UQ synthesis leading to depletion of the cellular UQ pool, and that epimastigotes, in order to survive, took up and utilized exogenous UQ_10_ instead of their physiological UQ, UQ_9_.

Notably, the supplementary effect of UQ_10_ was limited to a sub-lethal to lethal dose of each compound and was abrogated at the higher concentrations as shown in Fig 9. Because the polyisoprenoid chain of the UQ molecule varies in length depending on the species, the uptake of exogenous UQ with a different length isoprenoid chain (UQ_10_) cannot fully reconstitute the parasite UQ pool (UQ_9_), as reported before for *C*. *elegans* [52]. From a practical viewpoint, the limited rescue observed in the presence of supplemented UQ_10_ provides an important basis to consider trypanosomal UQ synthesis enzymes as drug targets. The infected parasites are not expected to survive by using the external UQ_10_ contained in the host human cells.

## Conclusions

Our findings shed light on the UQ biosynthesis as a promising drug target for CD. The DMQ_9_ accumulation by treatment with compound **1** (Figs 7 and 8) indicates that the oxazinoquinoline derivatives inhibited tCOQ7 and depleted the trypanosomal UQ pool, leading to death of the parasite. According to the study of other eukaryotes, the UQ head group is modified by multi-step enzymatic reactions, and the enzymes are presumed to have active centers with similar structures to catalyze similar quinone derivatives [17, 18, 53]. Therefore, we cannot rule out the possibility that our inhibitors cross-reacted and inhibited other trypanosomal UQ synthesis enzymes in addition to tCOQ7.

In this study, we indicated that the shortage of UQ pool in the protozoa was caused by the treatment with our inhibitor (Fig 8), and the treated epimastigotes were limitedly rescued by UQ_10_ supplementation (Fig 9). The supplementary effect was limited and abrogated by the higher concentrations of the inhibitors (Fig 9). It provides the evidence that *T*. *cruzi* is killed by the UQ depletion and the infected parasites would not be able to survive by using the external UQ_10_ contained in the host cells. We conclude that the UQ synthesis pathway of *T*. *cruzi* is a promising drug target for new CD treatment.

## Supporting information

S1 Fig Structures of active compounds in Table 1 and purity of compounds 1–3. Purity of compounds were determined by UPLC analysis (λ = 254 nm), performed on a Waters Acquity UPLC analytical system equipped with an ACQUITY UPLC BEH C18 column, 2.1 mm × 50 mm, 1.7 μm. Method: flux of 0.6 ml/min, 5−95% CH_3_CN in H_2_O + 0.1% TFA, total run time of 2 min.
(TIF)

S2 Fig Effect of compounds 1–3 on *T*. *cruzi* infection. The typical images of the host cells and amastigotes inside the host cells were shown. Each image was captured using an optical microscope equipped with a digital camera.
(TIF)

S3 Fig Compounds 1–3 killed the mammalian form of *T*. *cruzi*. Compounds were added to infected host cultures at the same time as the trypomastigote infection (black bars) or one day after the infection (gray bars). After 4 days, the infection rates were manually counted using three photos. The inhibition of infection (%) are indicated as mean ± standard deviation.
(TIF)

S4 Fig Low concentrations of compounds 1–3 increased the amount of hCOQ7 in HeLa cells. HeLa cells were inoculated and treated with compounds **1–3** for 3 days. The treated cells were harvested and lysed after wash with ice-cold PBS. The protein concentrations of the lysates were determined by the BCA protein assay kit, and 8 μg aliquots of each lysate were fractionated by SDS-PAGE and transferred to PVDF membranes. The membranes were blotted with each primary antibody, and developed using sheep anti-mouse IgG conjugated with horseradish peroxidase (NA931, GE Healthcare, Chicago, IL) and the ECL-Select reagent (for hCOQ7) or ECL-Prime reagent (for β-actin) (GE Healthcare). The anti-hCOQ7 mouse mAb (sc-376484) was purchased from Santa Cruz Biotechnology, Inc. (Santa Cruz, CA). The anti-β-actin mouse mAb (3700s) was from Cell Signaling (Danvers, CA).
(TIF)
