# Supplementary figures and images for: The ubiquinone synthesis pathway is a promising drug target for Chagas disease
Source: PLoS One. 2021 Feb 4;16(2):e0243855. doi: 10.1371/journal.pone.0243855 (PMC7861437; doi:10.1371/journal.pone.0243855)

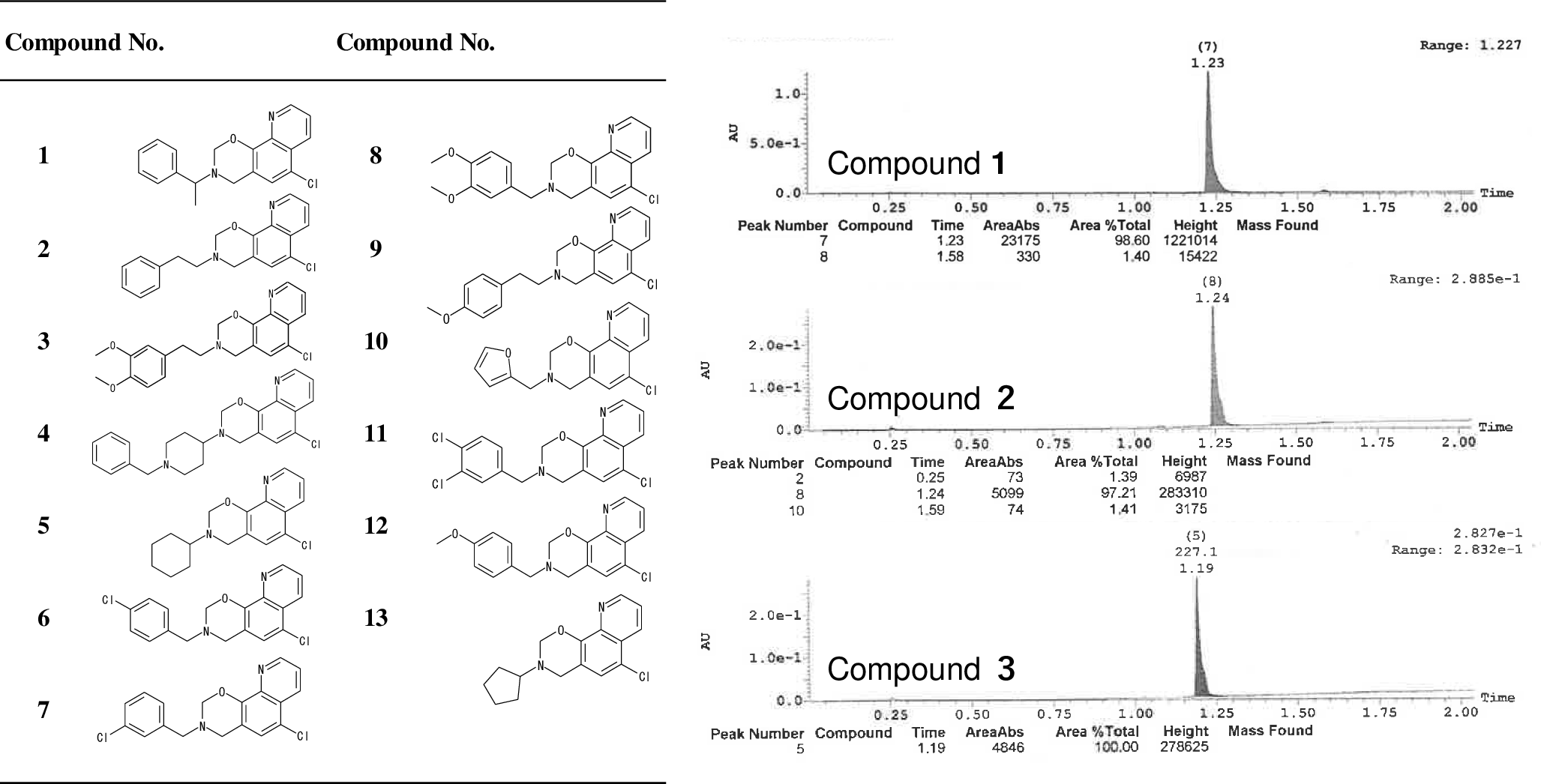

Supplement: S1 Fig — Purity of compounds were determined by UPLC analysis (λ = 254 nm), performed on a Waters Acquity UPLC analytical system equipped with an ACQUITY UPLC BEH C18 column, 2.1 mm × 50 mm, 1.7 μm. Method: flux of 0.6 ml/min, 5−95% CH3CN in H2O + 0.1% TFA, total run time of 2 min. (TIF) [file pone.0243855.s001.tif]

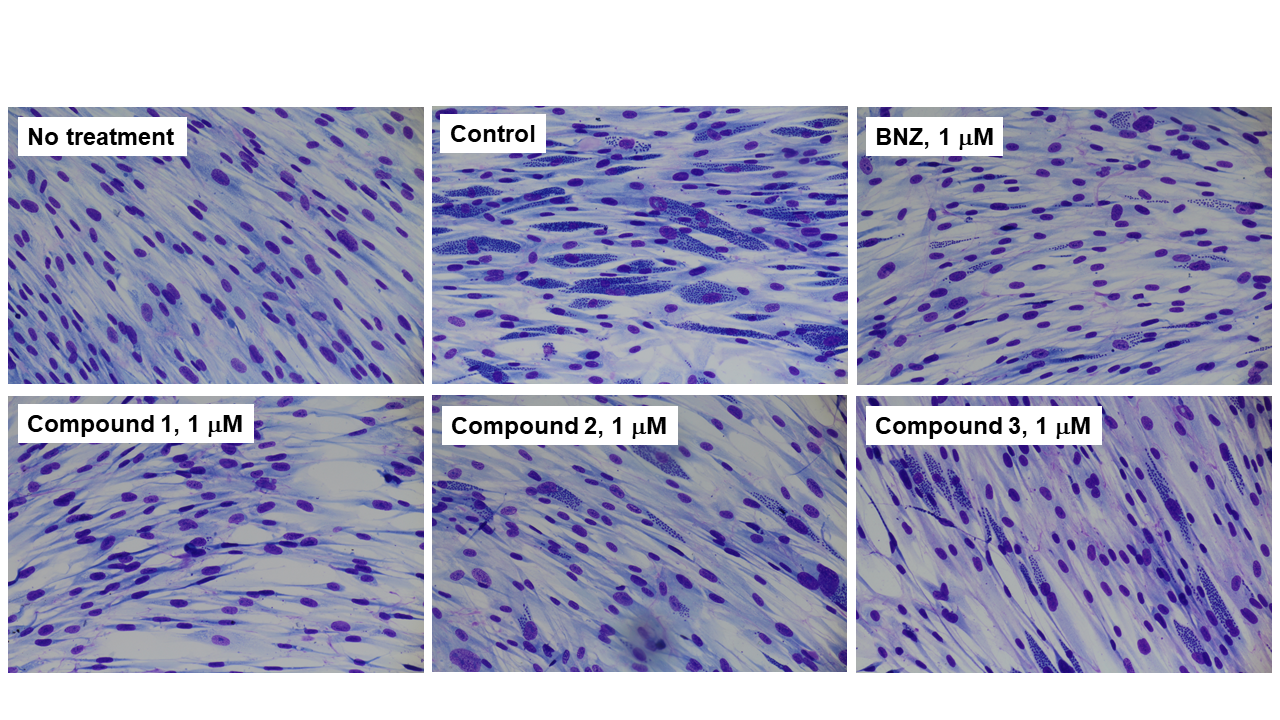

Supplement: S2 Fig — The typical images of the host cells and amastigotes inside the host cells were shown. Each image was captured using an optical microscope equipped with a digital camera. (TIF) [file pone.0243855.s002.tif]

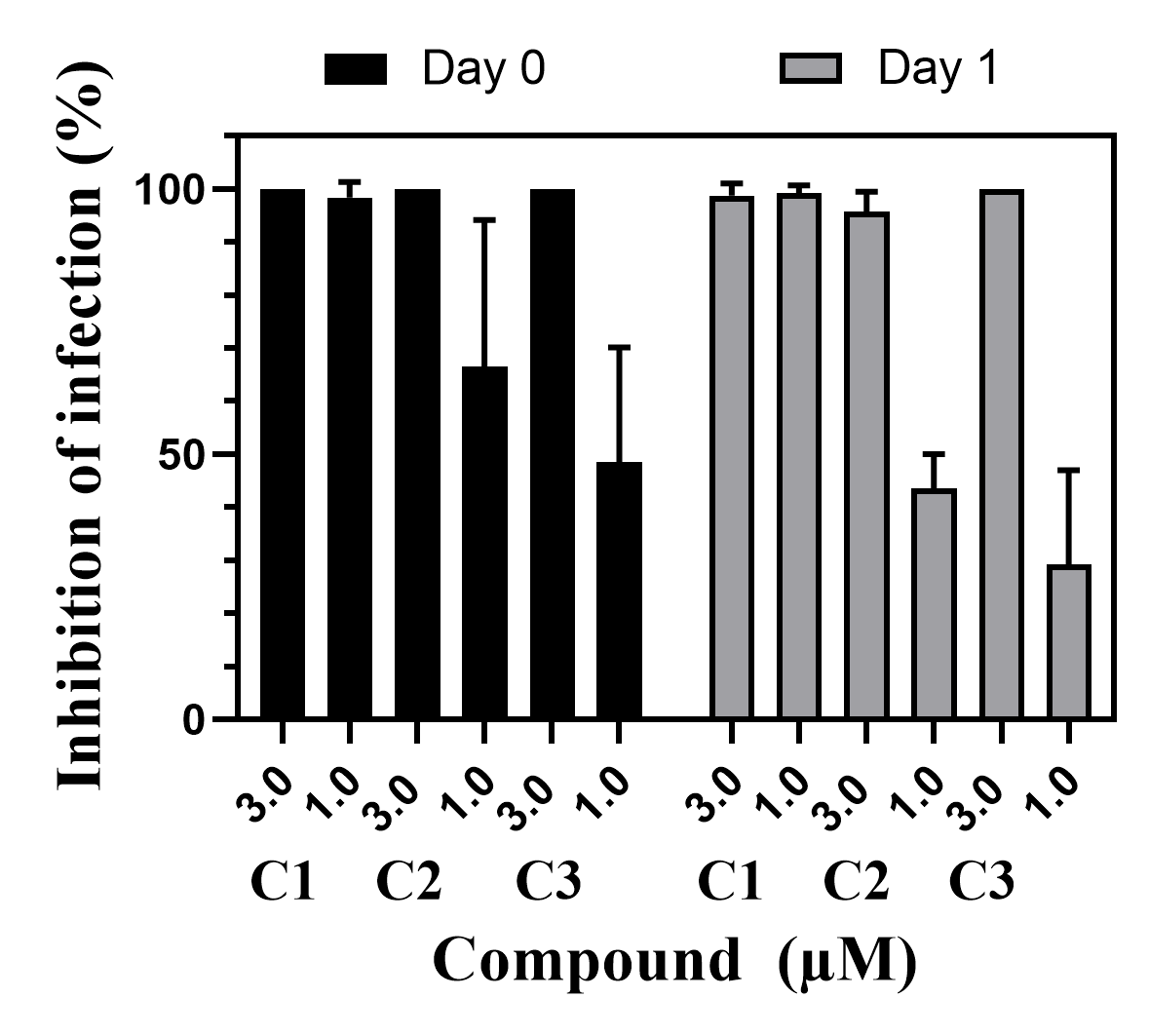

Supplement: S3 Fig — Compounds were added to infected host cultures at the same time as the trypomastigote infection (black bars) or one day after the infection (gray bars). After 4 days, the infection rates were manually counted using three photos. The inhibition of infection (%) are indicated as mean ± standard deviation. (TIF) [file pone.0243855.s003.tif]

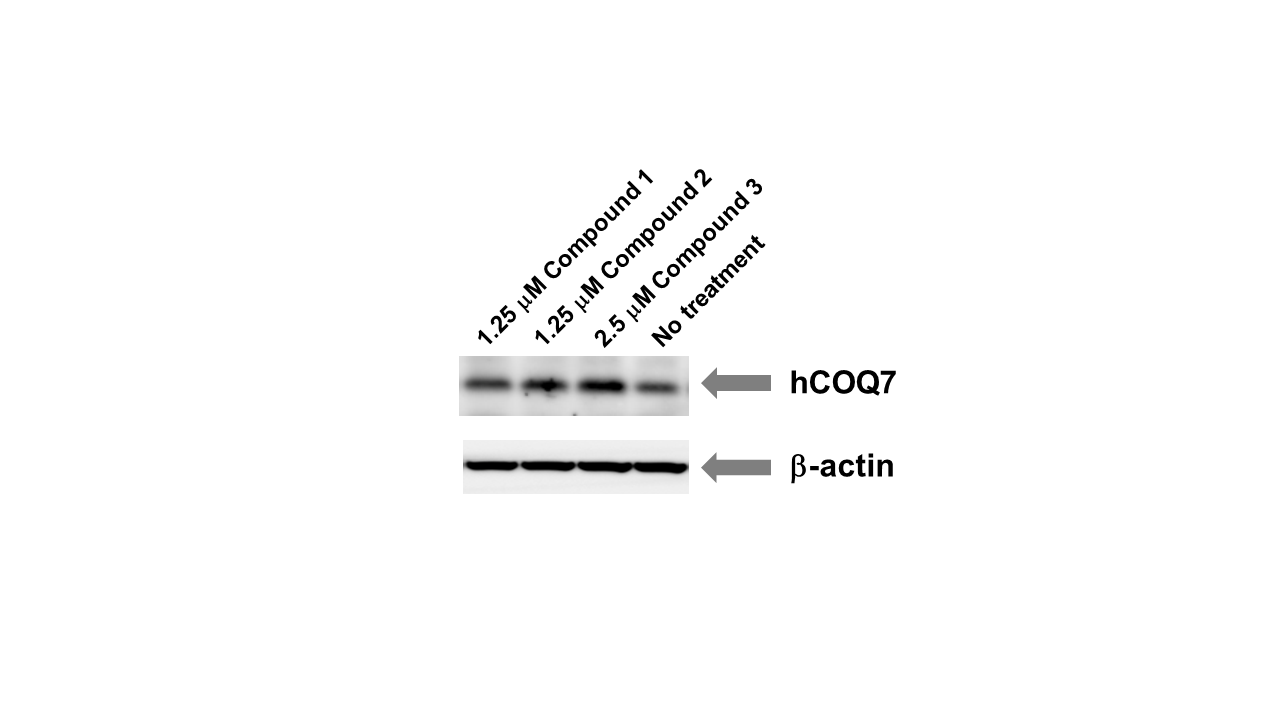

Supplement: S4 Fig — HeLa cells were inoculated and treated with compounds 1–3 for 3 days. The treated cells were harvested and lysed after wash with ice-cold PBS. The protein concentrations of the lysates were determined by the BCA protein assay kit, and 8 μg aliquots of each lysate were fractionated by SDS-PAGE and transferred to PVDF membranes. The membranes were blotted with each primary antibody, and developed using sheep anti-mouse IgG conjugated with horseradish peroxidase (NA931, GE Healthcare, Chicago, IL) and the ECL-Select reagent (for hCOQ7) or ECL-Prime reagent (for β-actin) (GE Healthcare). The anti-hCOQ7 mouse mAb (sc-376484) was purchased from Santa Cruz Biotechnology, Inc. (Santa Cruz, CA). The anti-β-actin mouse mAb (3700s) was from Cell Signaling (Danvers, CA). (TIF) [file pone.0243855.s004.tif]
